# Infection-responsive alternative splicing of *GhZDS* is associated with Verticillium wilt resistance in cotton

**DOI:** 10.1186/s12870-026-08981-1

**Published:** 2026-05-21

**Authors:** Hui Xi, Nuramina Sidik, Mingli Zhang, Li Liu, Xuwen Wang, Xinyu Li, Zhanjiang Tie, Sicheng Zhou, Yu Yu, Xuekun Zhang

**Affiliations:** 1 Xinjiang Academy of Agricultural and Reclamation Sciences, Shihezi, 832000 China; 2 College of Agriculture, Shihezi University, Shihezi, Xinjiang China

**Keywords:** Cotton, *Verticillium dahliae*, Alternative Splicing, Carotenoid Biosynthesis, *GhZDS*

## Abstract

**Background:**

Verticillium wilt, caused by the soil-borne fungus *Verticillium dahliae*, is a major disease limiting cotton production. Although transcriptional responses to *V. dahliae* infection have been widely studied, the temporal dynamics of alternative splicing (AS) and their potential association with disease resistance in cotton remain unclear.

**Results:**

To investigate infection-responsive AS regulation, we performed RNA-seq on root tissues of the resistant upland cotton cultivar JinKen 1775 (JK1775) and the susceptible cultivar Xinluzao 8 (Z8) at 12 h post-inoculation (12 hpi), 5 days post-inoculation (dpi), and 10 dpi with *V. dahliae.* Extensive AS reprogramming was detected in both cultivars, but their temporal patterns differed. In JK1775, AS events increased from 12 hpi to 5 dpi and then remained relatively stable at 10 dpi, whereas they continued to increase throughout the infection period in Z8. Differentially alternatively spliced (DAS) genes in JK1775 were enriched in several pathways, including carotenoid biosynthesis, pyrimidine metabolism, and phosphatidylinositol signaling. Among the candidate DAS genes, *GhZDS*, which encodes zeta-carotene desaturase, showed a stage-specific and infection-responsive splicing change in JK1775. RT-PCR and isoform-specific RT-qPCR confirmed the splicing change of *GhZDS*. Transient expression assays revealed distinct subcellular localization patterns for its two major isoforms. In addition, VIGS-mediated silencing of total *GhZDS* reduced resistance to *V. dahliae* in JK1775.

**Conclusions:**

Our study reveals distinct temporal AS responses in resistant and susceptible cotton during *V. dahliae* infection and identifies *GhZDS* as an infection-responsive, alternatively spliced candidate associated with Verticillium wilt resistance. These findings suggest that AS may contribute to carotenoid pathway-related defense responses in cotton. They also provide a basis for future isoform-specific functional studies.

**Supplementary Information:**

The online version contains supplementary material available at 10.1186/s12870-026-08981-1.

## Background

In eukaryotes, precursor mRNAs (pre-mRNAs) transcribed from genomic DNA undergo a series of processing steps to generate mature functional transcripts [1]. Alternative splicing (AS) is a key post-transcriptional regulatory mechanism that enables a single gene to produce multiple mRNA isoforms [2]. In the nucleus, the spliceosome catalyzes the selection of alternative splice sites (e.g., leading to exon skipping or intron retention), thereby generating distinct mRNA isoforms from a single pre-mRNA and greatly expanding the transcriptome and proteome complexity [3]. Genome-wide studies have shown that AS is widespread, affecting more than 90% of human genes [4], approximately 60% of *Arabidopsis thaliana* genes [5], and 40–60% of genes in major crops including cotton and rice [6]. In plants, AS is increasingly recognized as an important regulatory layer in development, hormone signalling, and stress responses [7–9]. Notably, pathogen infection can induce widespread reprogramming of host splicing patterns, positioning AS as a critical node in immune regulatory networks [10, 11].

Cotton is a globally important crop, serving as a primary source of natural fiber and vegetable oil [12]. Verticillium wilt, one of the most destructive diseases of cotton, is caused by the soil-borne fungus *V. dahliae* and severely reduces both yield and fibre quality [13]. This pathogen typically invades through the roots, colonizes the vascular system, and obstructs water conduction. This process results in characteristic symptoms, including leaf chlorosis, premature senescence, and plant death [14]. The persistence of *V. dahliae* in soil is enhanced by its long-lived microsclerotia, which can survive for decades. Disease incidence and severity are further exacerbated by agricultural practices like continuous cropping and by climate change [15]. Through prolonged co-evolution with vascular pathogens, cotton (a long-term cultivated tetraploid) has evolved a multi-layered defence system against persistent soil-borne infections [16]. This system encompasses robust capacities for cell wall biosynthesis and secondary metabolite production. Upon *V. dahliae* infection, cotton not only activates basal immunity but also reinforces xylem walls via lignin deposition and gel-like occlusions [17]. When pathogen colonization impedes water flow, cotton can modulate cellulose synthesis and stimulate new xylem formation to maintain hydraulic conductivity [18]. Hormonal regulation is also central to resistance, involving coordinated signalling of salicylic acid, jasmonic acid, ethylene, and strigolactone pathways [19]. Collectively, these adaptations suggest that cotton may deploy defense strategies that depend on species and tissue context compared with model plants.

Previous studies have shown that AS contributes to cotton resistance against *V. dahliae* [20]. In the highly Verticillium wilt-resistant wild diploid cotton species *Gossypium australe*, *V. dahliae* infection induces both the expression and AS of *GauSR45a* (*G. australe* serine/arginine-rich splicing factor 45a), a putative serine/arginine-rich splicing regulator involved in pre-mRNA processing. Silencing *GauSR45a* substantially reduces the splicing ratio of *V. dahliae*-induced immune-associated genes, including *GauBAK1* (*G. australe* BRI1-ASSOCIATED KINASE1) and *GauCERK1* (*G. australe* CHITIN ELICITOR RECEPTOR KINASE 1). Functional divergence among splice isoforms has also been reported in defence-related genes. For example, overexpression of *GauBAK1.1*, the short splice isoform of *G. australe* BRI1-ASSOCIATED RECEPTOR KINASE 1, significantly enhances cotton resistance to Verticillium wilt, whereas overexpression of the longer isoform *GauBAK1.2* does not. Together, these findings suggest that AS may modulate plant immunity by altering the composition and activity of key signalling components. Consistent with this idea, previous work also showed that *V. dahliae* infection induces a greater number of AS events in the resistant tetraploid cotton species *Gossypium barbadense* than in the susceptible tetraploid species *Gossypium hirsutum* [20].

AS also plays critical immune regulatory roles in other plant species. In *A. thaliana*, proper immune activation mediated by the receptor genes *RPS4* (RESISTANCE TO PSEUDOMONAS SYRINGAE 4) and *RPP7* (*RECOGNITION OF PERONOSPORA PARASITICA* 7) depends on specific transcript isoforms generated through AS and/or alternative polyadenylation [21, 22]. During infection with *Pseudomonas syringae* pv. *tomato* (Pst) DC3000, the splicing-associated complex component MAC3 (MOS4-ASSOCIATED COMPLEX subunit 3), a factor linked to pre-mRNA splicing and immune gene regulation, accumulates in the nucleus and promotes the expression of salicylic acid (SA)-associated immune marker genes, including *PR1*, *PR2*, *EDS1*, *ICS1*, *PR5*, and *EDS5* [23]. Moreover, pathogens can directly manipulate host RNA processing. The wheat stripe rust effector *Pst_A23* (*Puccinia striiformis* f. sp. *tritici* effector A23) binds conserved RNA motifs in pre-mRNAs and interferes with AS of immune-associated genes, such as *TaXa21-H* and *TaWRKY53*, thereby reducing their functional transcript levels and compromising host defence responses [10]. In *Nicotiana benthamiana*, the *Phytophthora* effector *PSR1* targets the splicing factor *PINP1* by binding its conserved C-terminal domains, leading to widespread alterations in AS patterns and enhanced pathogenicity of *Phytophthora sojae* [11].

The carotenoid biosynthesis pathway has been implicated in plant stress adaptation and defence regulation [24, 25]. In particular, 9-cis-epoxycarotenoids are important precursors for abscisic acid (ABA) biosynthesis, and *NCED1* (9-CIS-EPOXYCAROTENOID DIOXYGENASE 1) catalyzes a rate-limiting step in this process [26]. In *N. attenuata*, silencing the positive regulatory transcription factor *NaWRKY70* reduces ABA accumulation, increases stomatal conductance, and decreases the production of the ABA-regulated phytoalexin capsidiol, ultimately resulting in enhanced susceptibility to *Alternaria alternata* [27]. β-carotene also serves as a precursor for the plant hormone strigolactones (SLs), whose biosynthesis depends on key enzymes including CCD7 (CAROTENOID CLEAVAGE DIOXYGENASE 7), CCD8 (CAROTENOID CLEAVAGE DIOXYGENASE 8), and DWARF27 (D27 CAROTENOID ISOMERASE) [28]. In cotton, silencing *GhDWARF27*, *GhCCD7*, and *GhCCD8* compromises resistance to *V. dahliae*, whereas overexpression of *GhCCD8* enhances resistance [29, 30]. In addition, the *V. dahliae* effector *Vd06254* enhances *GhMYC3*-mediated repression of *GhCCD8*, further supporting the involvement of carotenoid-derived hormone pathways in cotton defence responses [31].

Although carotenoid biosynthesis and related hormone pathways (e.g., abscisic acid and strigolactone biosynthesis) have well-established roles in plant defense [26–31], how infection-responsive AS contributes to these responses remains largely unclear. In this study, we performed RNA-seq on resistant and susceptible upland cotton cultivars following *V. dahliae* inoculation. Our analysis characterized the dynamic AS landscape during infection. Together with experimental validation of the candidate gene *GhZDS*, these results support an association between infection-responsive alternative splicing of a carotenoid biosynthesis-related gene and Verticillium wilt resistance in cotton.

## Materials and methods

### Plant materials

Two upland cotton cultivars were used: the Verticillium wilt-resistant cultivar JinKen 1775 (JK1775) and the susceptible cultivar Xinluzao 8 (Z8). The seeds of both cultivars were provided by the Cotton Research Institute of Xinjiang Academy of Agricultural and Reclamation Sciences. Seeds were soaked in distilled water for 12 h, and then allowed to germinate on moist gauze for an additional 12 h. Germinated seeds were sown in sterile vermiculite and grown for 3 days. Seedlings were then gently washed and transferred to a Hoagland nutrient solution. They were maintained in a greenhouse under controlled conditions (16 h light/8 h dark, 25–28 °C) and grown for 11 days before pathogen inoculation.

### Pathogen inoculation

The defoliating strain of *V. dahliae* (V592), characterized by strong virulence, was kindly provided by Prof. Huang’s research group. Fungal stocks were revived on potato dextrose agar (PDA) plates and incubated for 3 days at 25 °C. Colonies were then transferred to Czapek-Dox liquid medium and cultured for 3 days at 25 °C with shaking at 180 rpm. The resulting spore suspension was filtered, and the spore concentration was adjusted to 1 × 10^7^ spores mL⁻¹ using sterile distilled water [32]. Seedlings were inoculated by dipping their roots in the spore suspension for 20 min and then returned to the greenhouse under the conditions described above. For each cultivar and time point, at least 15 seedlings were inoculated, constituting one biological replicate. The entire experiment was independently repeated three times. Root tissues were harvested at 12 hpi, 5 dpi, and 10 dpi, immediately frozen in liquid nitrogen, and stored at − 80 °C until RNA extraction and transcriptome sequencing.

### RNA-seq and read mapping

Total RNA was extracted from root samples collected at 12 hpi, 5 dpi, and 10 dpi using RNA extraction kit (Tiangen Biotech, China) according to the manufacturer’s instructions. For each combination of cultivar, treatment, and time point, three independent biological replicates were prepared and sequenced. Poly(A)-enriched mRNA libraries were constructed and sequenced on an Illumina HiSeq 2500 platform using a paired-end 150 bp (PE150) strategy, generating approximately 6 Gb of raw data per sample. Raw sequencing reads were quality-assessed using FastQC, followed by adapter trimming and filtration of low-quality/poly-N reads to obtain clean reads. Clean reads were aligned to the *G. hirsutum* TM-1 (ZJU-improved-v2.1-a1) reference genome using a splice-aware alignment approach. Mapping quality metrics were summarized for each library. The proportion of mapped reads ranged from 91.10% to 98.77%, and uniquely mapped reads accounted for 91.69% to 93.28% of clean reads (Supplementary Table S1). The resulting alignment files were utilized for subsequent AS analysis. To assess the reproducibility among biological replicates, we performed principal component analysis (PCA) based on normalized gene expression profiles. The results showed tight clustering of replicates within each condition, supporting the robustness of the RNA-seq dataset (Supplementary Fig. S1A).

### Identification of AS and differentially alternatively spliced events

The RNA-seq dataset analyzed in this study has been deposited in NCBI under BioProject accession PRJNA1121084. This dataset was previously reported in two published studies (PMID: 40243945; PMID: 39381515), which primarily focused on conventional transcriptome analyses and gene family characterization. In the present study, we re-analyzed the same RNA-seq reads, focusing on genome-wide AS dynamics, and performed additional experimental validations. AS events were identified and quantified using rMATS (v4.1.2) [33], which classifies AS events into five major types: skipped exon (SE), mutually exclusive exons (MXE), alternative 5′ splice site (A5SS), alternative 3′ splice site (A3SS), and retained intron (RI). Clean RNA-seq reads were aligned to the *G. hirsutum* reference genome TM-1 (ZJU-improved-v2.1-a1) using a splice-aware aligner, and the resulting coordinate-sorted BAM files, together with the corresponding gene annotation (GTF), were used as input for rMATS. For each comparison, BAM files from three biological replicates per condition were provided to rMATS for differential splicing analysis. In this study, SE and MXE were detected across the samples and were therefore included in subsequent analyses. For each AS event, rMATS estimates the exon inclusion level (ψ, percent spliced in) based on junction read counts. DAS events between conditions were identified based on a significant change in ψ (Δψ), with a threshold of |Δψ| > 0.1 and a false discovery rate (FDR) < 0.05. Genes with at least one DAS event were defined as DAS genes and are listed in Supplementary Table S2. These DAS genes were subsequently subjected to Gene Ontology (GO) and Kyoto Encyclopedia of Genes and Genomes (KEGG) pathway enrichment analyses using KOBAS software.

### Validation of DAS events and carotenoid pathway-related genes by RT-PCR and RT-qPCR

To validate RNA-seq-identified DAS events, root samples of the resistant cotton cultivar JK1775 were collected at 0, 12 hpi, 5 dpi, and 10 dpi after *V. dahliae* inoculation. Total RNA was extracted as described, and 1000 ng was reverse transcribed for first-strand cDNA synthesis. The resulting cDNA was diluted fivefold and used as template.

For RT-PCR, 1 µL of diluted cDNA was used per reaction; for RT‑qPCR, 20 µL reactions contained 2 µL of diluted cDNA. To validate the splicing pattern of *GhZDS*, isoform-specific forward primers were designed in the distinct 5′ UTR regions of *GhZDS-X1* and *GhZDS-X2*, respectively, along with a common reverse primer located in the shared coding sequence (CDS). *GhZDS-X1* and *GhZDS-X2* were amplified in separate RT-PCR reactions. Primer specificity was further confirmed by Primer-BLAST analysis. For additional DAS validation, *GhAFC3*, *GhPPD2*, and *GhXPO4* were selected. A common primer pair flanking the AS site was used for each gene, and isoforms were distinguished by product size. All RT‑PCR products were resolved on 2% agarose gels to confirm the predicted splicing patterns.

For RT-qPCR, isoform-specific primers for *GhZDS-X1* and *GhZDS-X2* were designed in the 5′ UTR. Primers for carotenoid pathway genes (*GhPSY2*, *GhCCD8b*, *GhNCED*3) were designed from NCBI sequences. *GhUB7* served as the internal control. Relative transcript levels were calculated using the 2^^−ΔΔCt^ method. Three independent biological replicates were analyzed per time point. All primer sequences used in this study are listed in Supplementary Table S3.

### Subcellular localization of *GhZDS* splice isoforms

To investigate the subcellular localization of the two *GhZDS* splice isoforms, the coding sequences (CDSs) of *GhZDS-X1* and *GhZDS-X2* (excluding the stop codon) were amplified and individually cloned into the pCAMBIA2300-GFP vector to generate C-terminal GFP fusion constructs. The recombinant plasmids were then introduced into Agrobacterium tumefaciens strain GV3101. Transient expression assays were performed in *N. benthamiana* leaves via Agrobacterium-mediated infiltration. At 48 h post-infiltration, fluorescence signals were observed using a Nikon AX R laser scanning confocal microscope. GFP fluorescence was excited at 488 nm, and emission signals were collected at 500–550 nm. Chlorophyll autofluorescence was excited at 640 nm, and emission signals were collected at 650–750 nm. Images were acquired from three independent infiltrated leaves. All images include a 20 μm scale bar.

### Evaluation of plant growth under mock conditions before pathogen inoculation

To determine whether silencing of *GhZDS* affected general plant growth before pathogen challenge, we evaluated the growth performance of TRV:*00* and TRV:*GhZDS* plants under mock conditions prior to *V. dahliae* inoculation. Measurements were taken at 10 days post-TRV infiltration, prior to pathogen inoculation. For each treatment, 15 plants were analyzed. The evaluated growth parameters included plant height, root length, fresh weight, and estimated leaf area. Representative photographs were also taken at the same time point.

### VIGS and functional validation of *GhZDS*

Virus-induced gene silencing (VIGS) was performed using a tobacco rattle virus (TRV)-based system in the cultivar JK1775. A 300-bp fragment from the CDS of GhZDS was amplified from cotton cDNA and cloned into the TRV2 vector to generate the TRV:*GhZDS* construct. The selected fragment lies within the shared coding region of the major *GhZDS* transcripts and is therefore designed to reduce total *GhZDS* transcript abundance rather than to selectively target a specific splice isoform. The empty TRV2 vector (TRV:*00*) served as a negative control. Both constructs were transformed into *Agrobacterium tumefaciens* strain GV3101. *Agrobacterium* cultures carrying TRV1 and either TRV:*GhZDS* or TRV:*00* were mixed at a 1:1 ratio and infiltrated into cotyledons of JK1775 seedlings using a needleless syringe [34]. Inoculated plants were kept in a growth chamber at 25 °C with a 16-h light/8-h dark photoperiod. Silencing efficiency was evaluated by RT-qPCR at 10 days post-TRV infiltration. Total RNA was extracted from the roots of TRV:*GhZDS* and TRV:*00* plants and reverse-transcribed into cDNA. Because the VIGS fragment was located in the shared coding region of *GhZDS*, it was expected to reduce the abundance of both major *GhZDS* isoforms. Therefore, in addition to total *GhZDS* expression, isoform-specific transcript levels of *GhZDS-X1* and *GhZDS-X2* were quantified using primers located in their distinct 5′ UTR regions. *GhUB7* was used as the internal reference gene, and relative expression levels were calculated using the 2^^−ΔΔCt^ method. For the disease assay, plants exhibiting strong *GhZDS* silencing were inoculated with V. dahliae strain V592 at 14 days post-TRV infiltration using the root-dipping method. Disease symptoms were evaluated at 14 dpi based on leaf wilting and vascular browning.

### Disease index evaluation and fungal biomass analysis

Disease severity was evaluated at 14 dpi using a disease index (DI). Each treatment consisted of three independent biological replicates, each containing 15 plants. Leaf symptoms were scored on a 0–4 scale (0, healthy; 4, severe wilting or defoliation), and the DI was calculated as follows: DI = [Σ (disease grade × number of plants in that grade) / (total number of plants × 4)] × 100 [35]. For fungal recovery assays, stem segments from above the cotyledonary node were harvested at 14 dpi, surface-sterilized, and placed on PDA medium to assess fungal growth. This assay was conducted with three independent biological replicates. Fungal biomass was quantified by RT-qPCR. Genomic DNA was extracted from stem tissues using the cetyltrimethylammonium bromide (CTAB) method [36] with minor modifications. Fungal DNA was quantified using the *V. dahliae*-specific primer pair ITS1-F and ST-VE1-R, with *GhUB7* as the internal reference gene [37]. Relative fungal biomass was calculated using the 2^^−ΔΔCt^ method.

### Statistical analysis

RT-qPCR data and disease assessments are presented as the mean ± standard deviation (SD) of three independent biological replicates. Plant growth parameters under mock conditions are presented as the mean ± SD of 15 plants per treatment. Statistical analyses were performed using SPSS software (v17.0). One-way analysis of variance (ANOVA) was used for comparisons among multiple groups, whereas comparisons between two groups were performed using Student’s t-test. Differences were considered statistically significant at *P* < 0.05 (*) and *P* < 0.01 (**). Graphs were generated using GraphPad Prism (v9.5). Heatmaps were produced using the online platform https://www.bioinformatics.com.cn for data analysis and visualization.

## Results

### Global dynamics of AS in resistant and susceptible cotton cultivars upon *V. dahliae* infection

**Fig. 1:**
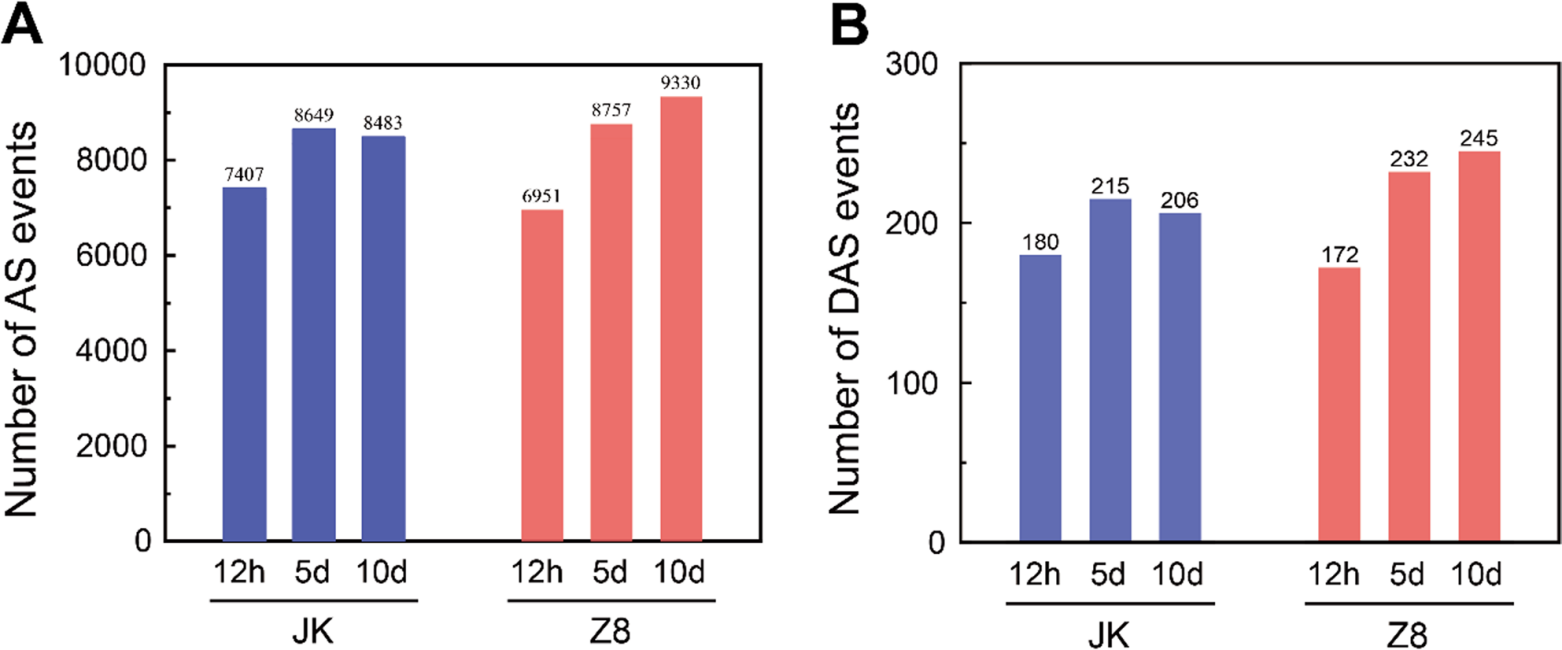
Dynamics of AS and DAS events in resistant and susceptible cotton upon V. dahliae infection. **A** Total number of AS events identified in JK1775 and Z8 at 12 hpi, 5 dpi, and 10 dpi. **B** Number of DAS events (|Δψ| > 0.1, FDR < 0.05) in each cultivar relative to mock-inoculated controls at the indicated time points.

Pathogen infection triggered extensive AS reprogramming in both cotton cultivars, with a substantial number of AS events detected at each time point. The resistant cultivar JK1775 displayed 7,407, 8,649, and 8,483 AS events at 12 hpi, 5 dpi, and 10 dpi, respectively. In contrast, the susceptible cultivar Z8 exhibited 6,951, 8,757, and 9,330 events at the corresponding time points (Fig. 1A). These data show an initial increase in AS events from 12 hpi to 5 dpi in both cultivars. However, their trajectories diverged markedly by 10 dpi. In JK1775, the number of detected AS events increased from 12 hpi to 5 dpi and then plateaued at 10 dpi, whereas in Z8, it rose continuously across all three time points. (Fig. 1A). Among the detected AS events, SE and MXE represented the predominant splicing types across all samples (Supplementary Fig. S1).

Comparative analysis against mock-inoculated controls identified DAS events (|Δψ| > 0.1, FDR < 0.05) (Fig. 1B). JK1775 contained 180, 215, and 206 DAS events at 12 hpi, 5 dpi, and 10 dpi, respectively, while Z8 contained 172, 232, and 245 DAS events. Notably, although the total number of DAS events was similar between the two cultivars at each time point, their temporal dynamics differed markedly. In JK1775, DAS events peaked at 5 dpi and then remained at a similar level, indicating an early and stabilized response. In contrast, Z8 showed a continuous, linear increase in DAS events throughout the infection period (Fig. 1B).

### KEGG pathway enrichment analysis of DAS genes

**Fig. 2:**
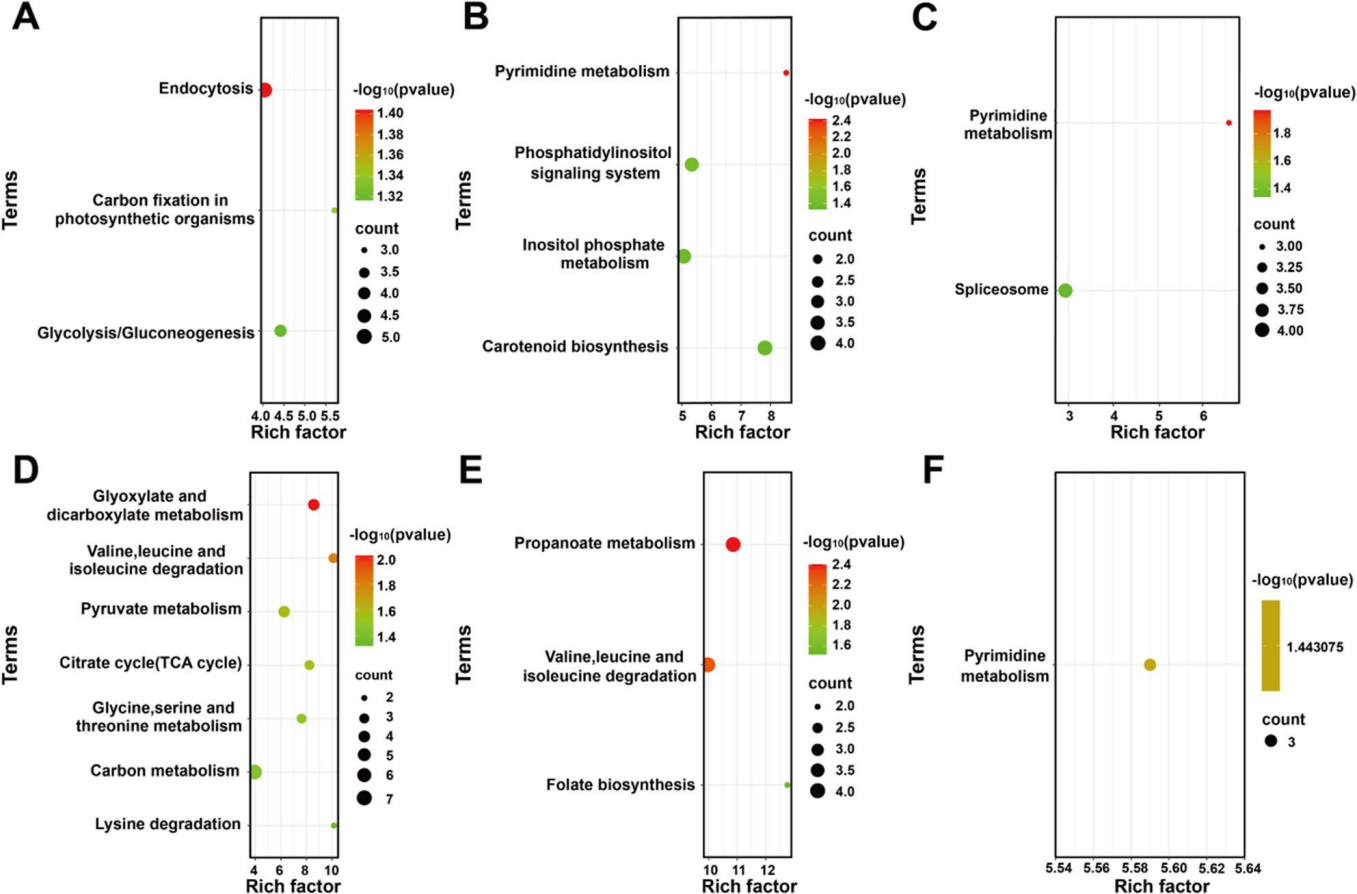
KEGG pathway enrichment analysis of DAS genes in JK1775 and Z8 following *V. dahliae* infection. Significantly enriched pathways (FDR < 0.05) for DAS genes in JK1775 (**A**–**C**) and Z8 (**D**–**F**) at 12 hpi, 5 dpi, and 10 dpi after inoculation

To elucidate the functional categories dynamically regulated by AS during infection, we performed KEGG pathway enrichment analysis on DAS genes in both cultivars across the three time points. This analysis revealed distinct and temporally shifting pathway enrichment profiles between the two cultivars (Fig. 2). At the early stage (12 hpi), DAS genes in JK1775 were enriched in endocytosis, glycolysis, and carbon fixation. In contrast, DAS genes in Z8 were enriched in core metabolic pathways including carbon metabolism, the tricarboxylic acid (TCA) cycle, and amino acid catabolism (Fig. 2A, D). By the intermediate stage (5 dpi), the enriched pathways in JK1775 shifted to pyrimidine metabolism, phosphatidylinositol signalling, and carotenoid biosynthesis, whereas Z8 was enriched in folate biosynthesis and amino acid degradation pathways (Fig. 2B, E). A striking divergence was observed at 10 dpi. In JK1775, DAS genes were significantly enriched not only in pyrimidine metabolism but also, notably, in the spliceosome pathway itself (Fig. 2C). Conversely, DAS genes in Z8 at 10 dpi showed minimal enrichment for the spliceosome and were instead enriched for broad metabolic and stress-responsive pathways (Fig. 2F). Collectively, these patterns suggest stage-dependent differences in the functional categories of DAS genes in JK1775, although the number of enriched genes in some pathways was limited. In contrast, the enrichment profile in Z8 was more static, dominated by sustained adjustments to core metabolism and general stress responses across time points. GO enrichment analysis at 5 and 10 dpi yielded complementary results (Supplementary Fig. S2).

### Comparative analysis of DAS genes between resistant and susceptible cultivars

**Fig. 3:**
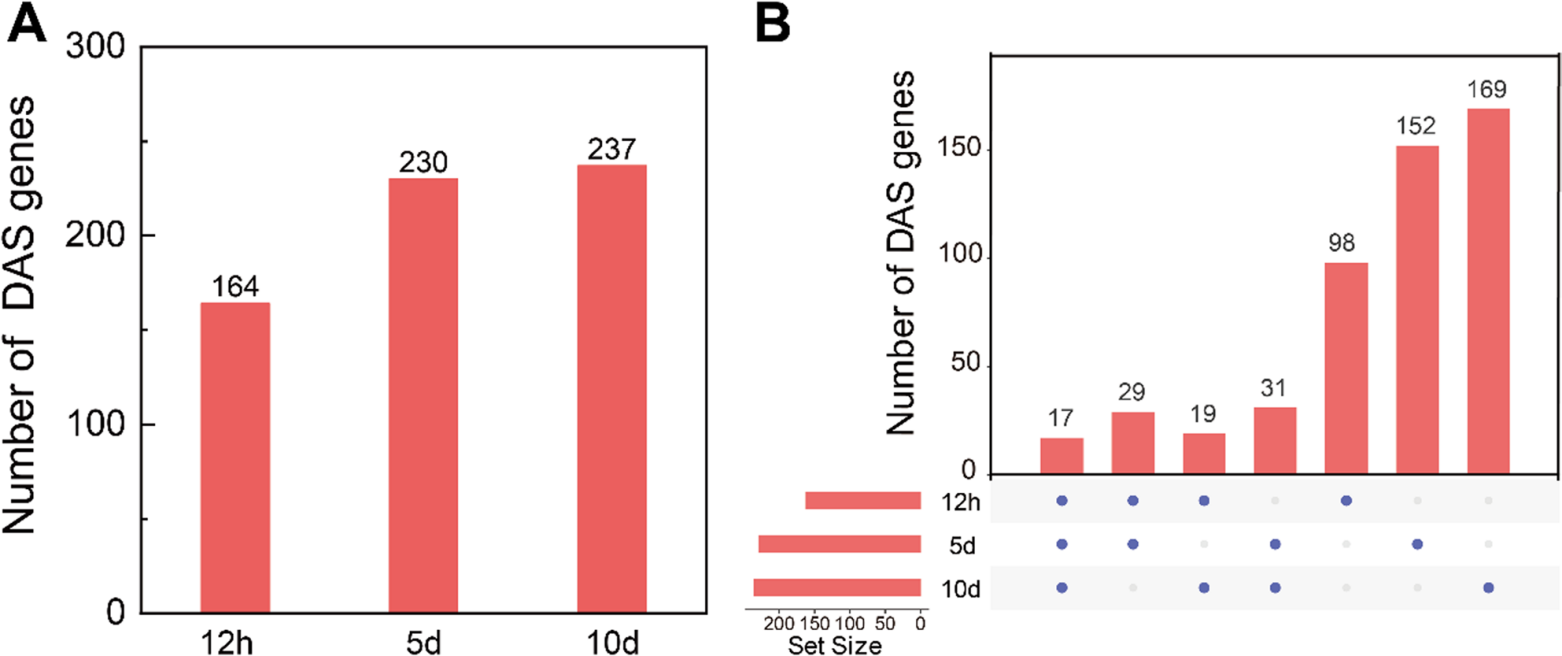
Number and overlap of DAS genes in JK1775 and Z8 after infection with *V. dahliae*. **A** The number of DAS genes that differ between JK1775 and Z8 at 12 hpi, 5 dpi, and 10 dpi. **B** UpSet plot showing the overlap of these cultivar-specific DAS genes across the three time points

Comparison of DAS genes between JK1775 and Z8 showed that the number of DAS genes increased from 12 hpi to 5 dpi and then changed only modestly from 5 to 10 dpi, from 164 at 12 hpi to 230 at 5 dpi and 237 at 10 dpi (Fig. 3A). This indicates that the divergence in splicing regulation between the resistant and susceptible genotypes intensified over the course of infection. An UpSet plot was used to visualize the temporal overlap among these between-cultivar DAS genes (Fig. 3B). The analysis showed limited overlap. Only 17 genes were common to all three time points, and pairwise overlaps were small (29 between 12 hpi and 5 dpi, 19 between 12 hpi and 10 dpi, and 31 between 5 and 10 dpi). However, the majority of DAS genes were stage-specific, with 98, 152, and 169 unique genes identified at 12 hpi, 5 dpi, and 10 dpi, respectively. These results demonstrate that the splicing divergence between the resistant and susceptible cultivars is predominantly stage-specific, reflecting dynamic and phase-dependent regulatory reprogramming rather than a static, constitutive difference.

**Fig. 4:**
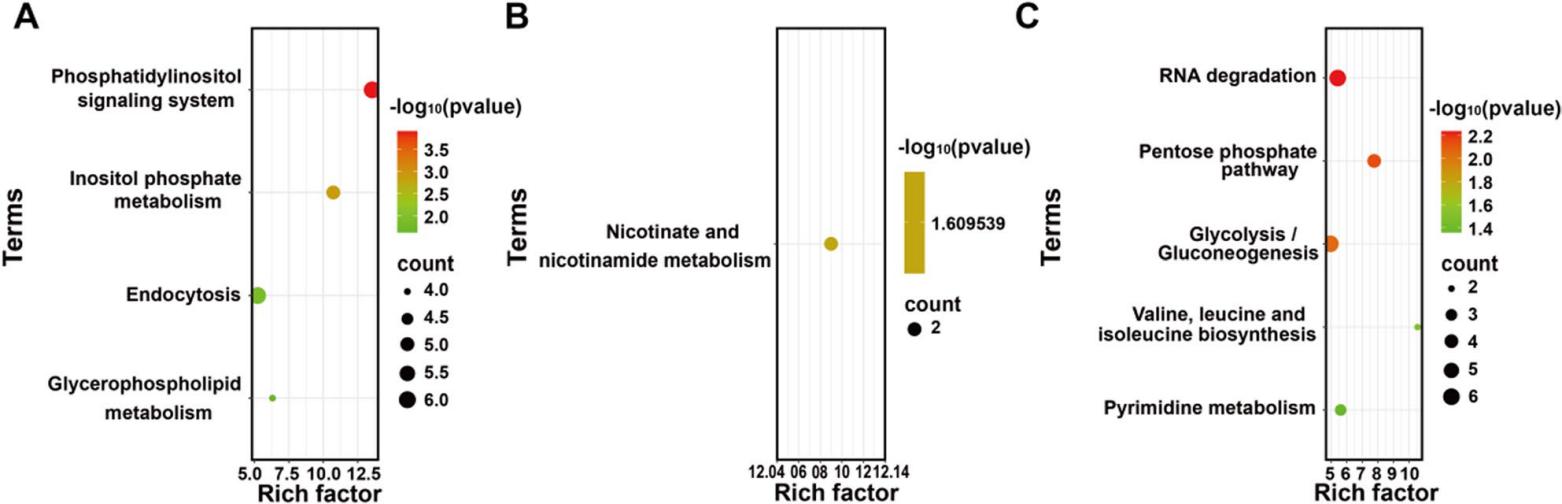
KEGG pathway enrichment of stage-specific DAS genes. Significantly enriched pathways (FDR < 0.05) for DAS genes identified between JK1775 and Z8 at (**A**) 12 hpi, (**B**) 5 dpi, and (**C**) 10 dpi

KEGG pathway analysis of the stage-specific DAS genes revealed distinct temporal shifts in the biological pathways differentially regulated by splicing between the two cultivars. At the early stage (12 hpi), DAS genes were enriched in pathways governing lipid signalling and membrane dynamics, including phosphatidylinositol signalling, inositol phosphate metabolism, glycerophospholipid metabolism, and endocytosis (Fig. 4A). By the intermediate stage (5 dpi), the enrichment profile became highly focused, with only nicotinate and nicotinamide metabolism showing significant enrichment (Fig. 4B). At the late stage (10 dpi), the spectrum of enriched pathways broadened again, encompassing diverse processes including valine, leucine and isoleucine degradation, pyrimidine metabolism, RNA degradation, and glycolysis/gluconeogenesis (Fig. 4C).

### Carotenoid pathway-related genes show coordinated infection-responsive changes in JK1775

**Fig. 5:**
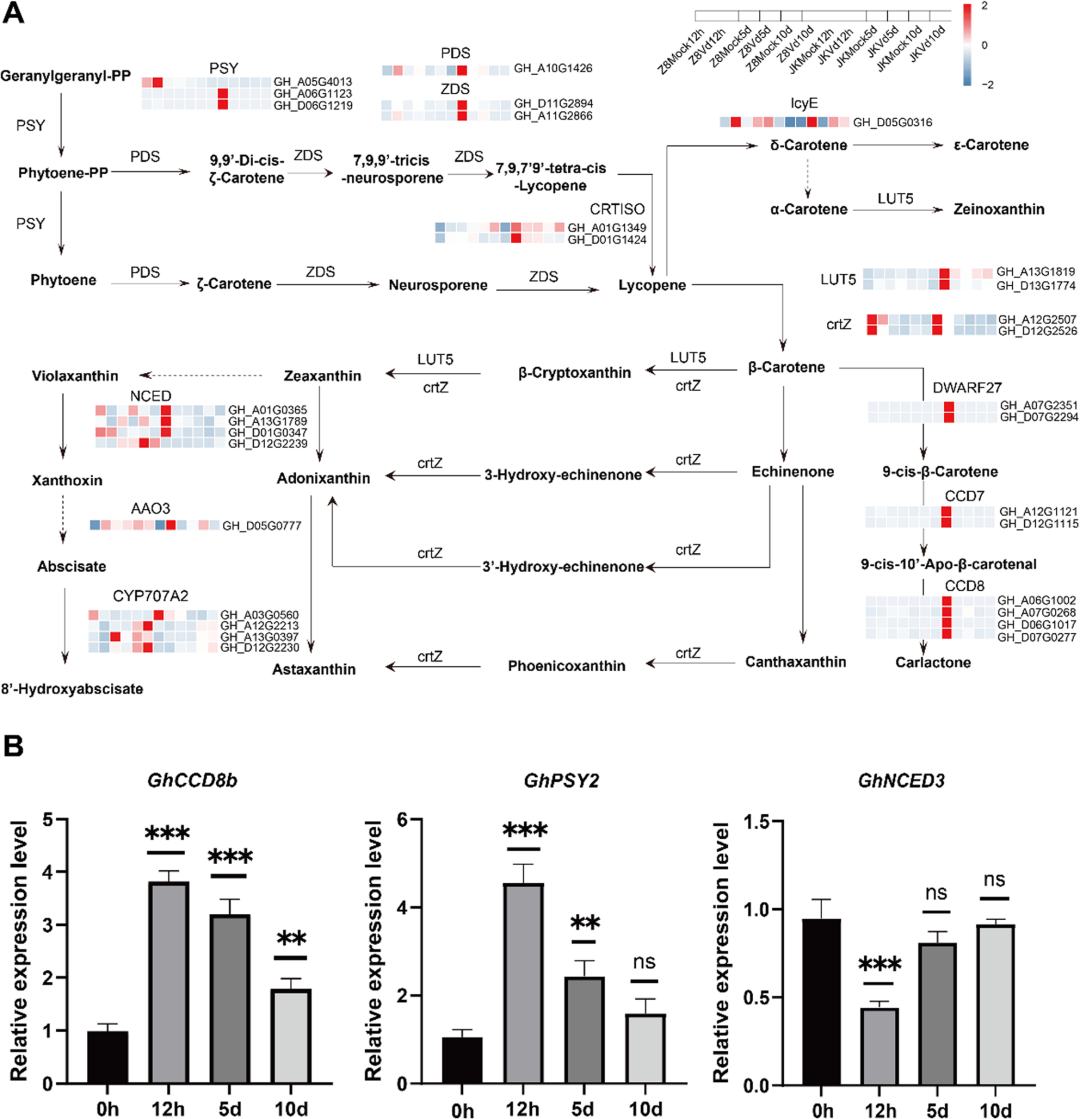
Infection-responsive changes in carotenoid pathway-related genes in JK1775 after *V. dahliae* inoculation. **A** Schematic overview of carotenoid metabolism and related downstream branches. Heatmaps show expression changes of representative genes at different time points after inoculation. Heatmap values represent log2 fold change (log2FC) relative to mock-inoculated controls. **B** RT-qPCR analysis of *GhCCD8b*, *GhPSY2*, and *GhNCED3* in JK1775 roots at 0, 12 hpi, 5 dpi, and 10 dpi. Expression levels were normalized to *GhUB7* and are shown relative to 0 hpi. Data are presented as mean ± SD of three biological replicates. Asterisks indicate significant differences compared with 0 hpi for each gene (**P < 0.01; ns, not significant)

KEGG enrichment analysis identified carotenoid biosynthesis as a candidate pathway associated with DAS genes at 5 dpi in JK1775 (Fig. 2B). We expanded the analysis to carotenoid-related pathways, including downstream ABA and SL branches, and observed infection-responsive changes in a subset of genes (Fig. 5A). Genes encoding *ZDS* and *PSY* exhibited both transcriptional changes and DAS signals. Genes in ABA-related branches (*NCED*, *CYP707A*) and SL pathway (*DWARF27*, *CCD7*, *CCD8*) also showed altered expression at specific time points. RT-qPCR analysis of *GhPSY2*, *GhCCD8b*, and *GhNCED3* in JK1775 roots (0, 12 hpi, 5, 10 dpi) showed that *GhPSY2* and *GhCCD8b* were rapidly induced at 12 hpi and then declined, whereas *GhNCED3* was downregulated at 12 hpi and recovered by 5 and 10 dpi (Fig. 5B). These results support the involvement of carotenoid pathway-related genes in the infection response of JK1775.

### Validation and molecular characterization of the *GhZDS* splicing event

**Fig. 6:**
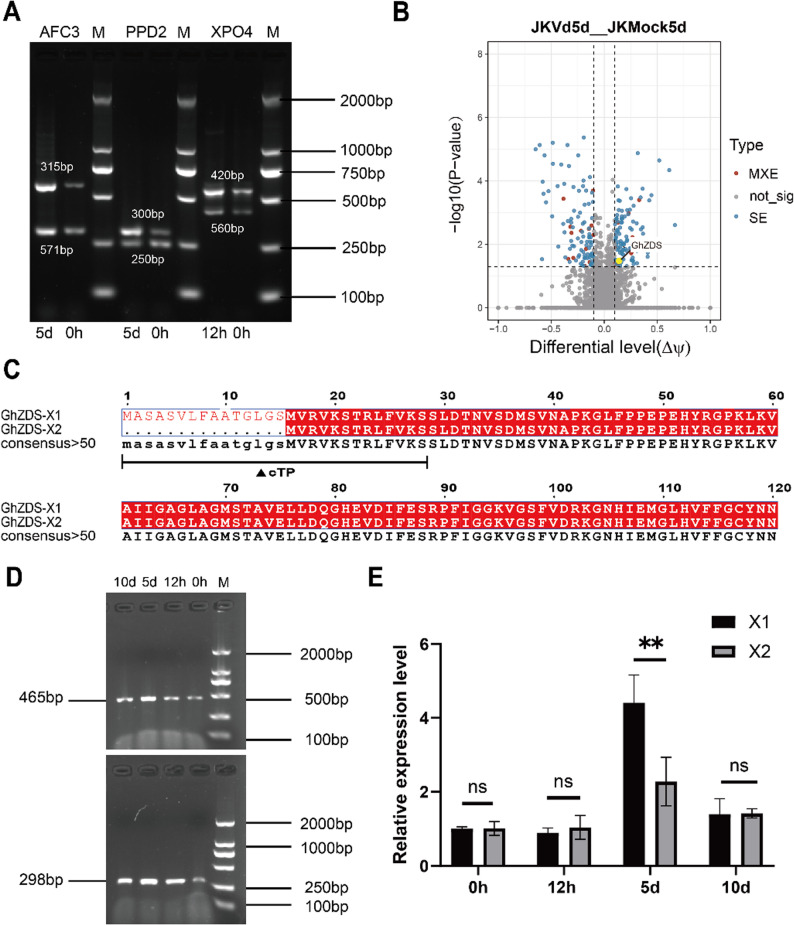
Validation and molecular characterization of the *GhZDS* splicing event. **A** RT-PCR validation of representative DAS genes (*GhAFC3*, *GhPPD2*, *GhXPO4*) in JK1775 at the indicated time points. **B** Schematic of the GhZDS locus and the infection-responsive AS event in JK1775 at 5 dpi. **C** Schematic of the predicted protein isoforms, highlighting the N-terminal truncation in X2. **D** RT-PCR validation of the two major *GhZDS* transcript isoforms using isoform-specific primers. *GhZDS-X1* and *GhZDS-X2* were amplified in separate reactions. **E** Isoform-specific RT-qPCR analysis of *GhZDS-X1* and *GhZDS-X2* in JK1775 at 0, 12 hpi, 5 dpi, and 10 dpi. Data are presented as mean ± SD of three biological replicates. Asterisks indicate significant differences between *GhZDS-X1* and *GhZDS-X2* at the same time point (**P < 0.01; ns, not significant)

To validate RNA-seq-identified DAS events, we selected three representative genes (*GhAFC3*, *GhPPD2*, *GhXPO4*) with large splicing differences (|Δψ| > 0.5) for RT-PCR analysis. The banding patterns matched rMATS-predicted splice forms, supporting the reliability of the DAS analysis (Fig. 6A). Among candidate DAS genes, *GhZDS* (zeta-carotene desaturase) attracted attention as a key enzyme in carotenoid biosynthesis that exhibited a pronounced infection-responsive splicing event in resistant JK1775 at 5 dpi (Fig. 6B). Comparison of the two major *GhZDS* isoforms (X1, X2) showed that alternative splicing altered the 5′ region, causing a 15‑amino‑acid truncation at the N‑terminus of X2 (Fig. 6C). This truncated region overlaps with the predicted chloroplast transit peptide of X1, but structural prediction indicated highly similar overall folds for both isoforms (RMSD = 0.17 Å), suggesting the truncation does not alter the conserved core structure (Supplementary Fig. S3).

To experimentally confirm the two *GhZDS* isoforms, we performed isoform-specific RT-PCR using forward primers targeting the distinct 5′ UTR regions of *GhZDS-X1* and *GhZDS-X2* together with a common reverse primer located in the shared coding region. *GhZDS-X1* and *GhZDS-X2* were amplified in separate RT-PCR reactions, and the expected products were detected for both isoforms (Fig. 6D). These results confirmed the presence of the two major *GhZDS* transcript isoforms. Isoform-specific RT-qPCR on roots collected at 0, 12 hpi, 5 dpi, and 10 dpi showed that relative expression levels of X1 and X2 differed significantly only at 5 dpi, with no difference at other time points (Fig. 6E). Together, these results indicate that *GhZDS* undergoes a stage‑specific, infection‑responsive splicing switch during *V. dahliae* infection in JK1775.

### Subcellular localization analysis of *GhZDS-X1* and *GhZDS-X2*

**Fig. 7:**
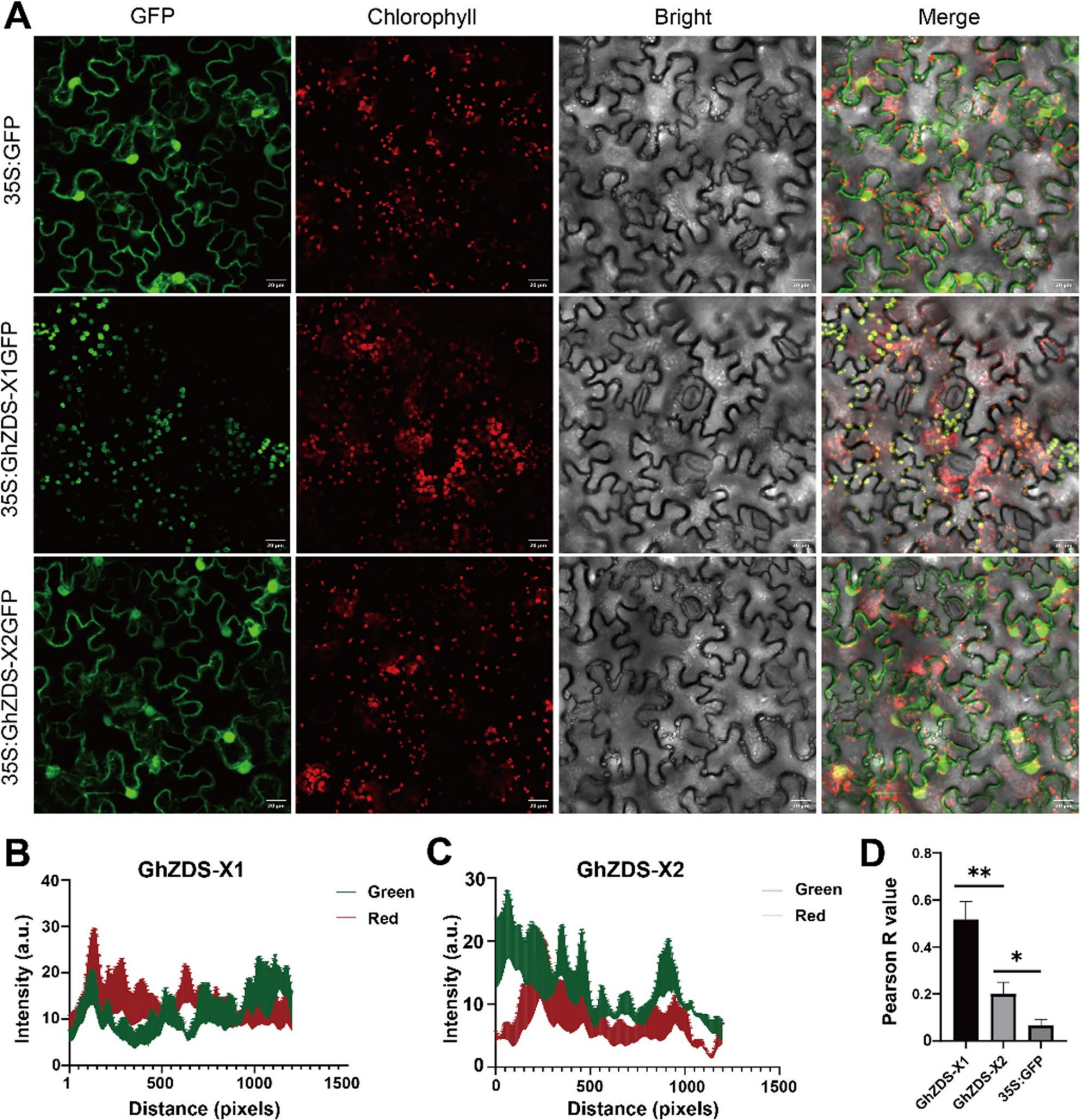
Subcellular localization of *GhZDS* splice isoforms in *N. benthamiana* leaves. **A** Confocal images showing GFP fluorescence, chlorophyll autofluorescence, bright-field, and merged signals in leaves transiently expressing 35S:GFP, 35S:GhZDS-X1-GFP, or 35S:GhZDS-X2-GFP. Scale bar = 20 μm. **B**, **C** Line-scan analysis of GFP and chlorophyll fluorescence in representative regions of three independent fields. Solid lines represent the mean intensity, and shaded areas indicate ± SD. **D** Pearson’s correlation coefficients between GFP fluorescence and chlorophyll autofluorescence calculated from three independent fields. Data are presented as mean ± SD. Statistical significance was assessed by Student’s *t*-test. Asterisks indicate significant differences between GhZDS-X1-GFP and GhZDS-X2-GFP, and between GhZDS-X2-GFP and 35S:*GFP*, as indicated by the horizontal brackets (*P < 0.05, **P < 0.01).

Since structural analysis suggested that the N-terminal difference between *GhZDS-X1* and *GhZDS-X2* may affect chloroplast targeting, we examined their subcellular localization by transient expression in *N. benthamiana* leaves. GFP fusion constructs of *GhZDS-X1* and *GhZDS-X2* were transiently expressed, and fluorescence signals were observed by confocal microscopy at 48 h post-infiltration. GhZDS-X1-GFP showed punctate fluorescence signals with stronger overlap with chlorophyll autofluorescence, whereas GhZDS-X2-GFP displayed broader cytoplasmic fluorescence and reduced overlap with chlorophyll autofluorescence compared with GhZDS-X1-GFP. To further evaluate the localization patterns, line-scan analysis of GFP and chlorophyll fluorescence was performed across representative regions from three independent confocal fields. The line-scan profiles showed greater overlap between GFP and chlorophyll signals for GhZDS-X1-GFP than for GhZDS-X2-GFP. Consistently, Pearson’s correlation coefficient analysis showed that GhZDS-X1-GFP had significantly higher overlap with chlorophyll autofluorescence than GhZDS-X2-GFP, whereas GhZDS-X2-GFP still showed higher overlap than the 35 S: GFP control. These results suggest that the two *GhZDS* isoforms differ in chloroplast-associated localization, consistent with the possibility that alternative splicing may affect the intracellular targeting efficiency of *GhZDS* (Fig. 7).

### Silencing of *GhZDS* compromises cotton resistance to *V. dahliae*

**Fig. 8:**
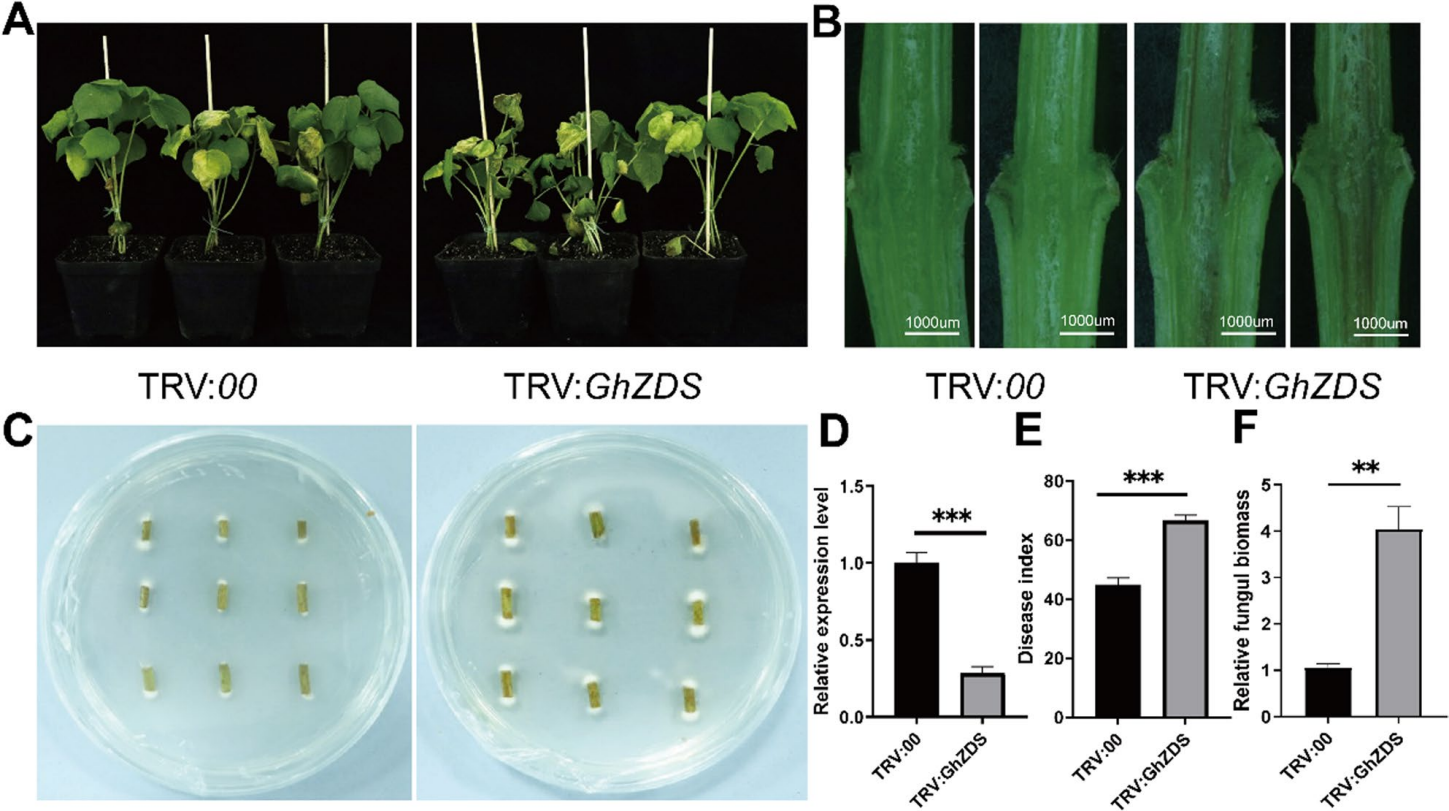
Functional analysis of *GhZDS* in Verticillium wilt resistance. **A** Phenotypes of *GhZDS*-silenced plants and control plants at 14 dpi after *V. dahliae* inoculation. **B** Vascular browning in stem longitudinal sections. **C** Fungal recovery culture from stems on PDA plates. **D** Relative total *GhZDS* expression levels in TRV:00 control and TRV:GhZDS plants. **E** DI of *GhZDS*-silenced and control plants at 14 dpi. **F** Relative fungal biomass in stems quantified by RT-qPCR. Data are mean ± SD (*n* ≥ 3). Asterisks indicate significant differences compared with the TRV:00 control (**P < 0.01; ns, not significant)

To investigate the role of *GhZDS* in cotton resistance to *V. dahliae*, we silenced *GhZDS* using VIGS. The VIGS fragment was located in the shared coding region of *GhZDS* and was therefore designed to silence total *GhZDS* rather than selectively target a single isoform. Isoform-specific RT-qPCR confirmed that both *GhZDS-X1* and *GhZDS-X2* transcript levels were significantly reduced in TRV:*GhZDS* plants compared with TRV:*00* controls (Supplementary Fig. S4D). Under mock conditions, no significant differences in plant height, root length, fresh weight, or estimated leaf area were observed between TRV:*GhZDS* and TRV:*0*0 plants (Supplementary Fig. S4A, B, C), indicating that *GhZDS* silencing does not noticeably affect normal plant growth. Following inoculation, *GhZDS*-silenced (TRV:*GhZDS*) plants developed significantly more severe disease symptoms than empty vector (TRV:*00*) controls. The DI was significantly higher in silenced plants, with symptoms including enhanced leaf chlorosis, wilting, and vascular browning at the cotyledonary node (Fig. 8A, B, E). Consistent with the visible symptoms, fungal biomass and pathogen recovery assays both confirmed significantly greater *V. dahliae* colonization in the stems of silenced plants. These results indicate that *GhZDS* contributes to cotton resistance against *V. dahliae*. The compromised resistance upon *GhZDS* silencing, together with the infection-responsive AS event identified here, suggests that *GhZDS* is required for effective Verticillium wilt resistance.

## Discussion

Verticillium wilt critically threatens global cotton production. While transcriptomic studies have extensively documented gene expression reprogramming in cotton upon *V. dahliae* infection, the role of AS in shaping defense responses remains poorly understood. The infection process of *V. dahliae* follows a defined temporal progression, with key stages including initial spore adhesion at approximately 12 hpi, hyphal establishment in epidermal cells around 5 dpi, and extensive vascular invasion by 10 dpi [38]. This temporal framework provides an opportunity to investigate stage-specific AS regulation during disease development. Accordingly, we performed a comparative transcriptome analysis of resistant and susceptible cotton cultivars with contrasting resistance levels to examine how AS may contribute to Verticillium wilt resistance.

Our global analysis revealed dynamic reprogramming of AS in both cultivars following *V. dahliae* infection. The total number of AS events increased from 12 hpi to 5 dpi in both lines, indicating progressive activation of splicing regulation during early infection. However, differences became apparent at the later stage. While AS event numbers stabilized in JK1775 at 10 dpi, they continued to increase in Z8. This pattern suggests that the resistant cultivar may exhibit a more coordinated and temporally controlled AS response, whereas the susceptible cultivar may undergo a more prolonged perturbation of splicing regulation. Previous studies have documented that pathogen infection can broadly perturb host RNA processing and splicing programs. For instance, TAL effectors from *Xanthomonas* target the rice splicing regulator *OsRBP11*, leading to extensive abnormal AS events [39]. In this context, the contrasting AS trajectories observed here may reflect distinct post-transcriptional response strategies between the two cotton cultivars.

The stage-specific pathway enrichment patterns further support this interpretation. At the early infection stage (12 hpi), DAS genes in JK1775 were enriched in endocytosis, glycolysis, and carbon fixation pathways. These processes may reflect rapid cellular reprogramming and energy mobilization associated with early defense responses. For instance, sakuranetin enhances rice resistance to blast disease by inhibiting clathrin-mediated endocytosis, highlighting the relevance of endocytic regulation in plant immunity [40]. In contrast, DAS genes in Z8 at 12 hpi were mainly enriched in basal metabolic pathways, including carbon metabolism, the TCA cycle, and amino acid catabolism, suggesting a different early response profile. The comparative analysis of cultivar-specific DAS genes further revealed that splicing divergence increased over time. Even at 12 hpi, DAS genes distinguishing the cultivars were enriched in lipid signaling pathways, which are central to early immune signal transduction and membrane remodeling [41]. This suggests that differences in early pathogen perception and signaling may contribute to the divergent outcomes of the two cultivars.

As infection progressed to the middle stage (5 dpi), JK1775 showed enrichment of DAS genes in pathways including phosphatidylinositol signaling, pyrimidine metabolism, and carotenoid biosynthesis. Among these, the carotenoid pathway is of particular interest because it is connected to carotenoid-derived hormone branches, including ABA and strigolactones, which have been implicated in plant stress responses and cotton resistance to *V. dahliae* [24, 29–31]. Our integrative analysis showed that genes associated with carotenoid-related pathways exhibited both transcriptional changes and DAS signals, and that these changes extended into downstream ABA- and strigolactone-related branches. Given that only a limited number of DAS genes were detected in some enriched pathways and that no carotenoid metabolites were directly quantified in this study, these observations should be interpreted as candidate associations rather than definitive evidence of pathway-wide reprogramming. Importantly, RT-PCR validation of representative DAS genes confirmed that the splicing changes predicted by rMATS reflected genuine transcript isoform variation during infection.

Within this carotenoid-related context, *GhZDS* emerged as a particularly informative case. *GhZDS* showed a stage-specific splicing event in the resistant cultivar, and RT-PCR confirmed the two major transcript isoforms. More importantly, isoform-specific RT-qPCR showed that the relative abundance of the two *GhZDS* isoforms differed significantly only at 5 dpi, with no significant difference at 0, 12 hpi, or 10 dpi. This result suggests that the *GhZDS* splicing switch is infection-responsive and stage-specific, rather than a constitutive difference between the two isoforms. Structural analysis further indicated that the two isoforms differ at the N-terminus. Transient subcellular localization assays showed that GhZDS-X1-GFP exhibited stronger overlap with chlorophyll autofluorescence, whereas GhZDS-X2-GFP displayed broader cytoplasmic fluorescence and reduced overlap with chlorophyll autofluorescence. These observations were further supported by line-scan and Pearson correlation analyses, indicating that the two isoforms differ in chloroplast-associated localization. This is consistent with previous evidence that AS can influence protein localization and metabolic function by generating isoforms with distinct subcellular localizations. For example, in *Arabidopsis*, AS of the Transthyretin-like (TTL) protein produces two isoforms: one contains a peroxisomal targeting signal (PTS2) and localizes to peroxisomes, whereas the other lacks this signal and localizes to the cytosol [42]. Together, these results suggest that infection-responsive AS may alter the relative abundance and localization of *GhZDS* isoforms during the critical 5 dpi stage.

Functionally, VIGS silencing of total *GhZDS* significantly compromised resistance to *V. dahliae*. In addition, *GhZDS* silencing did not cause detectable differences in general plant growth under mock conditions, supporting the interpretation that the enhanced disease susceptibility was not simply due to a general growth defect. Given the central role of *ZDS* in carotenoid biosynthesis, these findings suggest that AS-mediated modulation of *GhZDS* may influence carotenoid-associated defense responses, potentially including ABA- and strigolactone-related processes during infection [24, 29–31]. Consistent with the shared target region, isoform-specific RT-qPCR showed that both *GhZDS-X1* and *GhZDS-X2* were reduced in TRV:*GhZDS* plants. However, because the current VIGS construct targets shared *GhZDS* sequences rather than individual splice isoforms, the specific contribution of X1 versus X2 to resistance cannot yet be resolved. Therefore, our current data support an association between infection-responsive AS of *GhZDS*, altered isoform abundance, differential subcellular localization, and defense-related function. However, further isoform-specific functional analyses will be required to define the precise roles of the two isoforms in Verticillium wilt resistance.

At the late infection stage (10 dpi), DAS genes in JK1775 were enriched in pyrimidine metabolism and spliceosome-related pathways. This pattern suggests that the resistant cultivar may maintain active RNA-processing capacity during the later stages of infection. Notably, the involvement of spliceosome components is consistent with previous findings that disruption of the splicing factor *SR45a* in cotton compromises resistance to *V. dahliae* [20], underscoring the importance of intact splicing machinery in long-term defense.

## Conclusion

In summary, this study provides a time-resolved analysis of AS dynamics during *V. dahliae* infection in cotton roots and reveals distinct temporal AS patterns in the resistant cultivar JK1775 and the susceptible cultivar Z8. Our results identify *GhZDS* as an infection-responsive alternatively spliced gene associated with Verticillium wilt resistance. RT-PCR, isoform-specific RT-qPCR, and transient localization assays supported the infection-responsive splicing change of *GhZDS* and revealed distinct localizations of its two major isoforms. In addition, VIGS silencing showed that *GhZDS* contributes to resistance against *V. dahliae*. Together, these findings support a potential role for AS in carotenoid pathway-related defense responses in cotton. Further studies are required to determine the isoform-specific functions of *GhZDS* and the direct metabolic consequences of its AS.

## Supplementary Information

Supplementary Material 1.

Supplementary Material 2.

## Data Availability

The sequenced raw reads have been submitted to the National Center for Biotechnology Information (NCBI) under BioProject ID: PRJNA1121084. These data have been previously reported in two publications (PMID: 40243945; PMID: 39381515) and are re-analyzed here with a focus on alternative splicing.
