# Supplementary material for: Infection-responsive alternative splicing of GhZDS is associated with Verticillium wilt resistance in cotton
Source: BMC Plant Biol. 2026 May 21;26:1368. doi: 10.1186/s12870-026-08981-1 (PMC13466503; doi:10.1186/s12870-026-08981-1)
Supplement: Supplementary file 1 [file 12870_2026_8981_MOESM1_ESM.docx]

Supplementary Figure S1. AS profiling in cotton under *V. dahliae* infection. (A) PCA plot showing clustering of AS event profiles across samples. PC1 and PC2 explain 35.4% and 9.13% of variance, respectively. (B) Proportions of SE and MXE AS events in each sample group.

Supplementary Figure S2. GO analysis of DAS in JK1775 and Z8 after infection with *V. dahliae* strain V592. GO significantly enriched DAS genes in JK1775 at 5 dpi (A), 10 dpi (B). GO significantly enriched DAS genes in Z8 at 5 dpi (C), 10 dpi (D).

Supplementary Figure S3. Structural prediction and chloroplast transit peptide analysis of *GhZDS* splice isoforms. Predicted three-dimensional structures of *GhZDS-X1* and *GhZDS-X2*, gene structure comparison of the two transcript isoforms, and TargetP-2.0 prediction of chloroplast transit peptide probability.

Supplementary Figure S4. Growth assessment and isoform-specific silencing efficiency in TRV:*GhZDS* plants. (A) Comparison of plant height, root length, fresh weight, and estimated leaf area between TRV:*00* and TRV:*GhZDS* plants at 10 days after VIGS treatment. (B–C) Representative images of TRV:*00* and TRV:*GhZDS* plants grown under mock conditions. Data are shown as mean ± SD (n = 15). (Student’s t-test; ns, not significant). (D) Isoform-specific RT-qPCR analysis of *GhZDS-X1* and *GhZDS-X2* transcript levels in roots of TRV:*00* and TRV:*GhZDS* plants at 10 days post-TRV infiltration. Data are presented as mean ± SD. Asterisks indicate significant differences compared with TRV:*00* controls (**P* < 0.05, ***P* < 0.01; ns, not significant).
